# Intensive, interdisciplinary pain treatment in fibrous dysplasia/McCune–Albright syndrome

**DOI:** 10.1093/jbmrpl/ziag107

**Published:** 2026-06-29

**Authors:** Camryn Berry, Edin Randall, Julie Shulman, Shealyn O’Donnell, Catherine E Stewart, Jen Christofferson, Kevin Zirko, Ana Gallotto, Eleanor Powell, Zachary S Peacock, Amanda Cao, Ella Trumper, Boyu Ren, Michael Mannstadt, Ingrid A Holm, Navil Sethna, Jaymin Upadhyay

**Affiliations:** Department of Anesthesiology, Critical Care and Pain Medicine, Boston Children’s Hospital, Harvard Medical School, Boston, MA 02115, United States; Department of Psychiatry and Behavioral Sciences, Boston Children’s Hospital, Harvard Medical School, Boston, MA 02115, United States; Department of Physical Therapy and Occupational Therapy, Boston Children’s Hospital, Harvard Medical School, Boston, MA 02115, United States; Department of Physical Therapy and Occupational Therapy, Boston Children’s Hospital, Harvard Medical School, Boston, MA 02115, United States; Department of Psychiatry and Behavioral Sciences, Boston Children’s Hospital, Harvard Medical School, Boston, MA 02115, United States; Department of Psychiatry and Behavioral Sciences, Boston Children’s Hospital, Harvard Medical School, Boston, MA 02115, United States; Department of Physical Therapy and Occupational Therapy, Boston Children’s Hospital, Harvard Medical School, Boston, MA 02115, United States; Department of Physical Therapy and Occupational Therapy, Boston Children’s Hospital, Harvard Medical School, Boston, MA 02115, United States; Department of Physical Therapy and Occupational Therapy, Boston Children’s Hospital, Harvard Medical School, Boston, MA 02115, United States; Department of Oral and Maxillofacial Surgery, Massachusetts General Hospital, Harvard School of Dental Medicine, Boston, MA 02114, United States; Department of Anesthesiology, Critical Care and Pain Medicine, Boston Children’s Hospital, Harvard Medical School, Boston, MA 02115, United States; Endocrine Unit, Massachusetts General Hospital, Harvard Medical School, Boston, MA 02114, United States; Department of Psychiatry, McLean Hospital, Harvard Medical School, Belmont, MA 02478, United States; Endocrine Unit, Massachusetts General Hospital, Harvard Medical School, Boston, MA 02114, United States; Division of Genetics and Genomics, Boston Children’s Hospital, Harvard Medical School, Boston, MA 02115, United States; Department of Anesthesiology, Critical Care and Pain Medicine, Boston Children’s Hospital, Harvard Medical School, Boston, MA 02115, United States; Department of Anesthesiology, Critical Care and Pain Medicine, Boston Children’s Hospital, Harvard Medical School, Boston, MA 02115, United States; Department of Psychiatry, McLean Hospital, Harvard Medical School, Belmont, MA 02478, United States

**Keywords:** fibrous dysplasia, McCune–Albright syndrome, chronic pain, biopsychosocial model, intensive interdisciplinary pain treatment

## Abstract

Pain in fibrous dysplasia/McCune–Albright syndrome (FD/MAS) remains inadequately treated. The pilot study was aimed to determine the feasibility (ie, completion, retention, and acceptability) and to explore the effectiveness of an intensive interdisciplinary pain treatment (IIPT) using a biopsychosocial approach in patients with FD/MAS. Five patients (4 females and 1 male; 22-30 yr old) with FD/MAS, with baseline pain severity of 4+ (0-10 scale) were enrolled. A 3-wk intervention (4 d/wk; 3-4 h/d) integrating psychological, physical, and occupational therapies was administered to patients. Study measures included patient-reported outcomes at treatment admission (week 0), discharge (week 3), and follow-up (week 15). Physician Global Assessments (PGA) at weeks 0 and 3 were also collected. All 5 patients completed the 3-wk intervention and follow-up assessment and reported a high degree of acceptability, appropriateness, and feasibility of the intervention. Average pain severity decreased after treatment, with mean changes of −3.0 and −3.2 points at weeks 3 and 15, respectively. Patients demonstrated improvements in pain interference, external pain expression (measured with the pain behavior scale), and pain catastrophizing. Trends of decreased anxiety and depression were observed. Overall health-related quality of life improved at treatment discharge, which was consistent with the PGA (ie, +2 to +3) scores. This pilot study demonstrates that IIPT was feasible and showed initial clinical benefit across multiple dimensions in a small cohort of patients with FD/MAS. Larger-scale, controlled trials are needed for further validation.

## Graphical Abstract

**Graphical Abstract:**
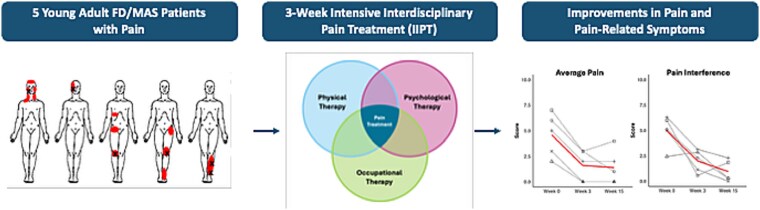

## Introduction

Fibrous dysplasia (FD) is a rare skeletal disorder caused by somatic, heterozygous gain-of-function variations (R201H or R201C) in the *GNAS* gene, leading to mosaic activation of the alpha subunit of the stimulatory G protein in bone.^1–4^ Fibrous dysplasia manifests as lesions characterized by the replacement of normal bone and marrow with fibro-osseous tissue. Fibrous dysplasia may occur in isolation or as part of McCune–Albright syndrome (MAS), which includes endocrinopathies and hyperpigmented skin macules. Fibrous dysplasia lesions can involve a single bone (monostotic FD) or multiple bones (polyostotic FD) and affect the craniofacial and axial-appendicular skeleton. Lesions are associated with skeletal deformities, fractures, and the development of chronic pain.^3^^,^^5–7^

Pain is the most common symptom of FD/MAS, reported in up to 81% of adults.^8–11^ Patients with craniofacial FD lesions may present with headaches, migraines, and/or orofacial pain, while lesions in the axial-appendicular skeleton may lead to musculoskeletal pain.^12–14^ Notably, pain severity does not correlate with the amount of skeletal involvement or lesion activity, as evaluated by radiotracer uptake on, for example, ^18^F-NaF PET/CT or bone turnover markers.^7^^,^^9^^,^^11^^,^^15–18^

Pain in FD/MAS remains difficult to effectively manage, with existing therapeutic strategies yielding variable outcomes across patients.^2^^,^^8^^,^^10^^,^^19–21^ Treatments used to mitigate pain in FD/MAS include surgical interventions or pharmacological therapies, such as bisphosphonates, denosumab, and more recently, burosumab.^22–27^ However, the efficacy or durability of these modalities with respect to pain relief across patients with FD/MAS is inconsistent.^10^ Adverse effects (eg, marked bone-turnover rebound with hypercalcemia following discontinuation of denosumab) can also limit therapeutic use.^6^^,^^28^ Patients with FD/MAS also use acetaminophen and non-steroidal anti-inflammatory drugs, often intermittently, but their therapeutic value is largely untested in FD/MAS populations.

The biological mechanisms underlying FD-related pain remain incompletely understood. Despite the development of different animal models of FD, it is only recently that a potential pain phenotype of these animal models has been explored.^29^ Initial studies demonstrated behavioral deficits in FD mice, and subsequent work confirmed nociceptive behaviors that were partially reversible with analgesics, suggesting the development of pain-like responses as lesions progress.^5^^,^^30^^,^^31^ Analyses of tissue samples from FD mice implicate several potential mechanisms, including the presence of nociceptive and sympathetic nerve fibers, increased osteoclast activity, and elevated levels of inflammatory cytokines, chemokines, and nerve growth factor.^5^^,^^32^^,^^33^ Expression of nerve growth factors, including brain-derived neurotrophic factor (BDNF), has been reported in mouse and human FD tissues.^34–36^ Notably, although sensory nerve fibers are present within FD lesions, there has been no evidence of nerve sprouting or neuroma formation in either mouse models or human FD tissue samples. This indicates that pain sensation in FD/MAS may occur in the absence of morphological changes of peripheral innervation and implicates inflammatory, neurochemical, or central mechanisms.^34^

Emerging evidence suggests that pain in FD/MAS is shaped not solely by lesion-level biological mechanisms but also by centrally mediated and/or biopsychosocial factors, including possible central sensitization.^11^^,^^18^^,^^37^^,^^38^ Prior work has shown that the severity of pain experienced by FD/MAS patients is associated with factors, such as pain catastrophizing and self-reports of depression.^17^ Neuroimaging-based examination of structural (gray and white matter) and functional central nervous system properties implicated circuits that regulate sensorimotor and emotional aspects of pain. These findings are similar to the complex relationships between biological and psychological factors observed in persons with chronic pain with and without FD/MAS. This complexity aligns with the biopsychosocial model, which conceptualizes chronic pain as an integrated experience influenced by (neuro-)biological pathology, psychological processes, and social context.^39^^,^^40^ Such factors and interactions can facilitate a chronic pain state and underscore the need for interventions that simultaneously target multiple, extra-skeletal dimensions of pain in FD/MAS. Biopsychosocial approaches that emphasize education and self-management approaches are recommended for persons with chronic pain and widely supported by evidence in adult and pediatric populations.^41–43^

Guided by prior pain phenotyping studies of patients with FD/MAS, we sought to evaluate the feasibility and explore the effectiveness of intensive interdisciplinary pain treatment (IIPT) for improving biopsychosocial outcomes (eg, pain, physical health, emotional health, and overall quality of life) in this disease.^17^^,^^18^^,^^44–46^ This pilot feasibility study explored the clinical impact of 3-wk of IIPT in young adults with FD/MAS. This program integrates an interdisciplinary team of physicians, psychologists, physical therapists, and occupational therapists to improve patient’s quality of life. The goal of IIPT is to enhance functioning and participation in daily activities despite the presence of pain. Key interventions include pain neuroscience education, exercise, psychotherapy (with cognitive behavioral therapy [CBT] and acceptance and commitment therapy [ACT] components), and medication management. Intensive interdisciplinary pain treatment supports persons with chronic pain by providing tools that enable them to (1) understand biopsychosocial factors that may contribute to pain episodes, (2) develop prevention strategies, and (3) employ active coping skills. The objective of this small-scale, hypothesis generating clinical study was to evaluate the feasibility and clinical utility of IIPT to inform the development of future controlled trials targeting pain and related dysfunction in FD/MAS.

## Materials and methods

### Study participants

This study was approved by the Boston Children’s Hospital (BCH) IRB (IRB-P00047474) in 2024. Patient recruitment was aided through local physician referrals and the FD/MAS Alliance and Registry. Potential participants were given an overview of study procedures and subsequently provided written informed consent. Study enrollment and evaluation took place between January 2025 and June 2025. Diagnosis of FD/MAS was confirmed through review of medical history and imaging (ie, ^18^F-sodium fluoride PET/CT [^18^F-NaF PET/CT], CT, or MRI) as well as in accord with prior established guidelines.^4^ Additional patient inclusion criteria were male or female, 18-30 yr of age, and a self-reported average pain rating of 4 or greater on a 0-10 scale for at least 3 mo prior to screening caused by or associated with an FD lesion. Patients were excluded if they had active injury or impending fractures, untreated substance misuse (excluding cannabis), significant cognitive impairment that precluded participation in treatment, severe psychiatric comorbidity including active suicidal ideation or psychosis, or functional neurologic disorders such as uncontrolled seizures or recurrent syncope that would interfere with safe participation. Patients were not excluded based on current or prior use of bisphosphonates or denosumab, and concomitant pain medications were permitted and supervised by a pain physician.

### Pain treatment overview

**Figure 1:**
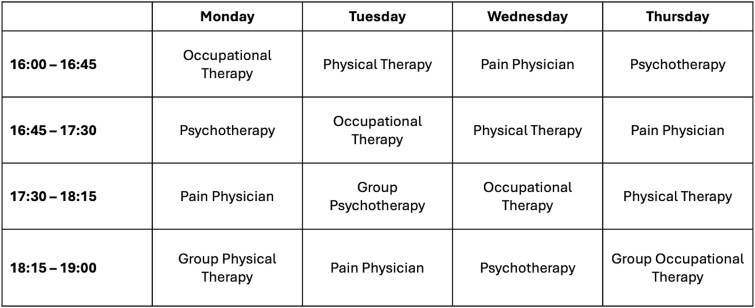
Sample weekly schedule. Treatment occurred four days per week for 3-4 h per day, where each day included individual or group sessions of physical therapy, occupational therapy, and psychotherapy. Patients also had a clinical session with a pain physician.

Participants completed an IIPT program in an outpatient setting at the Young Adult Pain Rehabilitation Center at BCH. This program focuses on IIPT in patients between 18 and 30 yr of age. Treatment was administered in 3- to 4-h, 4 days per week, over a 3-wk period. A sample weekly schedule is provided in Figure 1. At treatment admission, an individualized treatment plan was developed collaboratively by the clinical team for each participant. On each treatment day, participants engaged in treatment sessions with a member of the interdisciplinary team (~45-min sessions/discipline). Each interdisciplinary team included a pain physician, psychologist, physical therapist, and occupational therapist. Sessions were conducted in individual and/or group formats depending on treatment goals or clinical needs.

#### Psychological therapy

Cognitive behavioral therapy and ACT frameworks were implemented. At treatment onset, a psychologist and the patient completed a psychological assessment and co-developed an individualized biopsychosocial formulation incorporating FD/MAS-related factors and chronic pain mechanisms to guide the intervention. Therapeutic content emphasized the bidirectional relationship between psychosocial factors and pain-related disability. Individual and group-based psychological therapy included psychoeducation about chronic pain, teaching physiological self-regulation strategies, cognitive skills training, and an emphasis on a values-based life. Psychoeducation was delivered verbally and through structured worksheets and videos. Targeted treatment goals included: (1) increasing insight into biopsychosocial factors affecting pain experiences, (2) building confidence in managing pain and illness-related challenges, (3) identifying and addressing unhelpful thoughts associated with chronic pain, and (4) establishing strategies to balance illness management with engagement in valued life activities and relationships. Recommendations for post-IIPT treatment (psychotherapy, neuropsychological evaluation, activity scheduling, and FD/MAS social support) was also provided.

#### Physical therapy

Physical therapy (PT) focused on improving functional independence and participation in meaningful activities using a psychologically informed approach. Upon admission, each participant was evaluated by a physical therapist to determine the individual’s impairments, functional limitations, participation restrictions, and unifying PT diagnoses. Evaluation included a detailed interview and PT examination relevant to the presenting complaints. Interventions emphasized a self-management approach and prioritized safe progression of exercise (aerobic exercise, strength training, balance, and flexibility training), gradual exposure to symptom-provoking activities while engaging in active coping tools. Pain neuroscience education was also emphasized. Passive PT modalities were avoided (eg, manual therapy techniques). Comprehensive home exercise programs were prescribed for all participants to perform once daily and included aerobic, flexibility, and strength components.

#### Occupational therapy

Targeted treatment areas addressed functional limitations associated with chronic pain, sensory intolerances (eg, tactile, visual, and auditory sensitivities), cognitive difficulties, and restrictions in daily role participation. The primary goals of occupational therapy (OT) were to improve independence in self-care, activities of daily living, academic or work performance, and engagement in leisure activities. Interventional components included training to enhance attention during cognitively demanding tasks, strategies to support self-regulation in stimulating environments, and gradual weaning from adaptive equipment when appropriate. For participants with sensory sensitivities (eg, scalp allodynia and photophobia), therapists implemented individualized progressive desensitization programs to normalize responses to everyday sensory stimuli.

### Outcome measures

A comprehensive questionnaire battery (see also Supplemental Material) probing (1) pain and sensitization (Brief Pain Inventory [BPI], painDETECT, Central Sensitization Inventory, Headache Impact Test, PROMIS: Pain Behavior, Pain Catastrophizing Scale, Fear of Pain, and Chronic Pain Acceptance); (2) mental health (PROMIS: Anxiety, PROMIS: Depression, and NIH Toolbox Perceived Stress); and (3) quality-of-life (Self-Efficacy for Managing Chronic Disease, WHO Disability Assessment Schedule, and EuroQol 5-Dimensions 5-Levels) was completed by each patient at treatment admission (week 0, prior to engagement with any provider), treatment discharge (week 3), and mid-term follow-up (week 15). At treatment discharge, each patient was also asked 3 open-ended questions to gauge their perspective on participating in the IIPT program: (1) What were the most helpful parts of this program? (2) What parts of the program do you wish had been different? (3) What were the most important things that you learned from the program? Three measures of fidelity, Acceptability of Intervention Measure, Intervention Appropriateness Measure, and Feasibility of Intervention Measure,^47^ were also administered at treatment discharge.

The Physician Global Assessment (PGA) was independently completed by a pain physician (NS; Physician 1) and an endocrinologist (IH; Physician 2) to track patient progress and treatment response (see Table S1). Physician 1 met with each patient on each day of the program, while Physician 2 only evaluated each patient for a 60-min period at weeks 0 (prior to treatment onset) and 3 (after treatment discharge).

Serum samples obtained at treatment admission and discharge were analyzed in batch for calcium, phosphorus, albumin, alkaline phosphatase (ALP), C-terminal telopeptide (CTX), and procollagen type 1 N-terminal propeptide (P1NP) to assess skeletal metabolic status at the 2 timepoints.

### Statistical analysis

The impact of IIPT on patient reported outcome measures described in Supplemental Material across weeks 0, 3, and 15 was analyzed. Kendall’s coefficient of concordance (W) was performed to determine effect sizes. The Page test for ordered alternatives was also used to assess trends across evaluation timepoints. The Page test is a nonparametric method designed to detect directional change across repeated measures and is well suited for small sample sizes, as it does not assume normality. All statistical analyses were performed in R (version 4.3.1).

## Results

### FD/MAS patient overview

**Figure 2:**
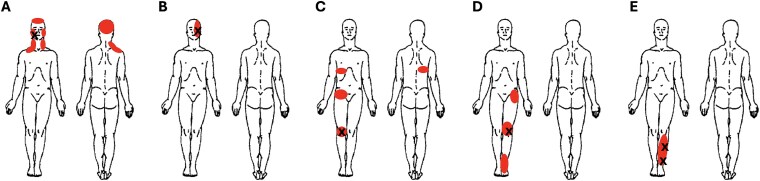
Self-reported pain locations in patients with FD/MAS. Study participants marked the locations (shown in red) where they experienced chronic pain at week 0; for all patients, these locations corresponded to sites of known FD lesion involvement. The location causing the most severe pain is indicated with an X. Skeletal burden scores are reported in Table S2. (A) Patient 1: 24-yr-old female diagnosed at age 16 with polyostotic FD in the right orbit, maxilla, zygoma, and mandible. (B) Patient 2: 27-yr-old female diagnosed at age 23 with monostotic FD in the left maxilla. (C) Patient 3: 22-yr-old female diagnosed at age 6 with McCune-Albright syndrome. She had extensive FD affecting the craniofacial and axial-appendicular skeleton. (D) Patient 4: 25-yr-old female diagnosed at age 5 with polyostotic FD in the left femur, left tibia, mandible, and occipital bone. (E) Patient 5: 30-yr-old male diagnosed at age 12 with monostotic FD in the left tibia.

All 5 patients (mean ± SD age, 25.6 ± 3.0 yr; 4 females and 1 male) completed the 3-wk interdisciplinary pain treatment program consisting of PT, OT, psychotherapy, and pain medicine visits delivered 4 d per week, followed by a 15-wk follow-up assessment. There were no reports of adverse events for any enrolled patient with FD/MAS. The mean age of diagnosis was 12.4 ± 7.4 yr. Two patients had monostotic FD, 2 patients had polyostotic FD, and 1 patient was diagnosed with MAS (Figure 2, Table S2). Two patients had FD affecting only the craniofacial skeleton (patients 1 and 2), 1 patient had FD affecting only the axial-appendicular skeletal (patient 5), and 2 patients had FD affecting both the craniofacial and axial-appendicular skeleton (patients 3 and 4). Only patient 1 was receiving treatment with anti-resorptive treatment (zoledronate) during the study. In Supplemental Material, case descriptions are provided for each study participant. Here, we provide (1) patient overviews, (2) baseline clinical assessment, and (3) individualized treatment plans.

### Patient perspectives on treatment experience

**Figure 3:**
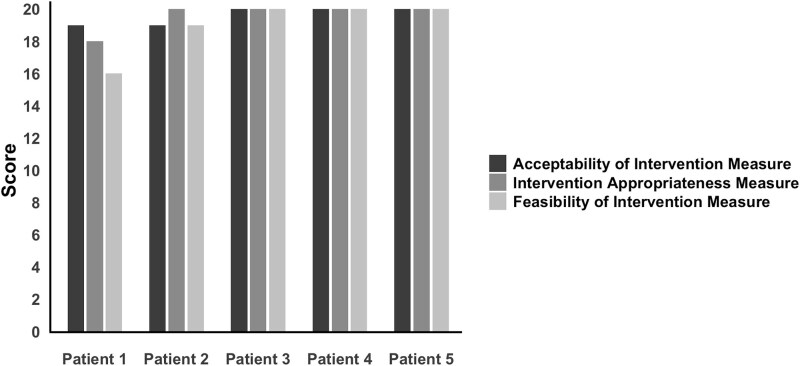
Patient ratings of the acceptability, appropriateness, and feasibility of the pain treatment. At week 3, each patient completed 3 measures of fidelity (Acceptability of Intervention Measure, Intervention Appropriateness Measure, and Feasibility of Intervention Measure). All patients noted a high level of satisfaction with the pain treatment program, which corroborated responses to open-ended questions (Table S3).

The Acceptability of Intervention Measure, Intervention Appropriateness Measure, and Feasibility of Intervention Measure (0-20 scale) measured patients’ satisfaction with the IIPT program at week 3 (Figure 3). A high-level of acceptability and utility was quantified across measures, which also corroborated input received from patients during open-ended questions about the clinical utility of the treatment program and potential improvements (Table S3).

### Characterization of pain treatment effect (patient-reported outcome)

**Figure 4:**
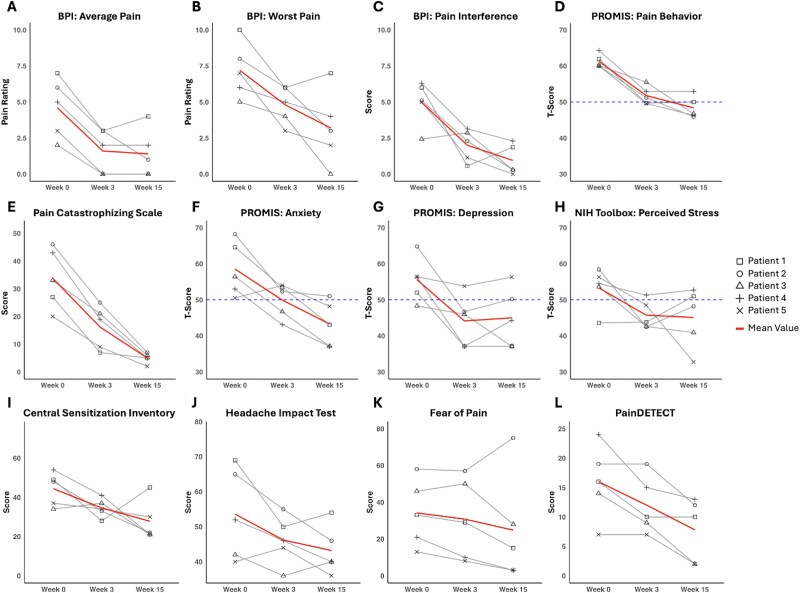
Patient reported outcomes at weeks 0, 3, and 15. (A-C) Using the Brief Pain Inventory (BPI), the severity of average pain, worst pain, and pain interference showed significant decreases between week 0 (treatment admission), week 3 (treatment discharge), and week 15 (mid-term follow-up). The impact of pain and maladaptive thoughts about the patients pain as measured by the PROMIS: pain behavior (D) and pain catastrophizing scale (E), respectively, also decreased. The severity of anxiety (F) and depression (G) was reduced at weeks 3 and 15 with rebound occurring in some patients at week 15 for both measures. (H) A trend of reduced perceived stress occurred at week 3 and 15. (I) Central Sensitization Inventory scores showed significant decreases from week 0 to week 3 and were maintained for most patients at week 15. (J) Headache Impact Test scores decreased at weeks 3 and 15, with the largest improvements observed in patients with craniofacial FD. (K) Fear of Pain Questionnaire scores generally decreased by week 15, indicating reduced pain-related fear. (L) Neuropathic-like pain quality, as measured by PainDETECT, improved; all patients screened as unclear or negative for neuropathic-like pain at week 15. The dashed blue line in panels D, F, G, and H represents a T-score of 50, which is the reference value for the general U.S. population for PROMIS measures.

Clinical questionnaire scores (group mean ± SD) at weeks 0, 3, and 15 and change in scores between timepoints are reported in Table 1. Pain severity and pain interference as assessed with the BPI^48^ (BPI; 0-10 scale), decreased between weeks 0 and 15, with only 1 participant demonstrating a partial rebound at week 15 (Figure 4). Average pain severity at treatment admission and discharge, compared to pain ratings from a historical FD/MAS cohort, is shown in Figure S1.

**Table 1:** Clinical questionnaire scores.

**Outcome measure**	**Week 0** **(mean ± SD)**	**Week 3** **(mean ± SD)**	**Week 15** **(mean ± SD)**	**Change** **(week 0 vs week 3)**	**Change** **(week 0 vs week 15)**	**Change** **(week 3 vs week 15)**	**Kendall’s W effect size**
**Brief Pain Inventory: average pain**	4.6 ± 2.1	1.6 ± 1.5	1.4 ± 1.7	−3.0	−3.2	−0.2	0.88
**Brief Pain Inventory: pain right now**	2.8 ± 1.6	0 ± 0	0.6 ± 1.3	−2.8	−2.2	+0.6	0.95
**Brief Pain Inventory: worst pain**	7.2 ± 1.9	4.8 ± 1.3	3.2 ± 2.6	−2.4	−4.0	−1.6	0.84
**Brief Pain Inventory: least pain**	2.8 ± 2.8	0.6 ± 0.9	0.6 ± 1.3	−2.2	−2.2	0.0	0.57
**Brief Pain Inventory: pain severity composite score**	4.4 ± 1.6	1.8 ± 0.9	1.5 ± 1.4	−2.6	−2.9	−0.3	0.84
**Brief Pain Inventory: pain interference composite score**	5.0 ± 1.5	2.0 ± 1.1	0.9 ± 1.0	−3.0	−4.1	−1.1	0.64
**PainDETECT**	16.0 ± 6.3	12.0 ± 4.9	7.8 ± 5.4	−4.0	−8.2	−4.2	0.86
**Central Sensitization Inventory**	44.4 ± 8.5	34.6 ± 4.8	27.8 ± 10.3	−9.8	−16.6	−6.8	0.64
**Headache Impact Test**	53.6 ± 13.1	46.2 ± 7.1	43.2 ± 7.0	−7.4	−10.4	−3.0	0.52
**PROMIS: pain behavior**	61.3 ± 1.9	51.8 ± 2.5	48.4 ± 3.0	−9.5	−12.9	−3.4	0.83
**Pain Catastrophizing Scale**	33.8 ± 10.8	16.2 ± 7.8	5.0 ± 1.9	−17.6	−28.8	−11.2	1
**Fear of Pain**	34.2 ± 18.2	30.8 ± 22.4	24.8 ± 29.9	−3.4	−9.4	−6.0	0.36
**PROMIS: anxiety**	58.5 ± 7.6	49.9 ± 4.8	43.3 ± 6.3	−8.6	−15.2	−6.6	0.84
**PROMIS: depression**	55.6 ± 6.2	44.1 ± 7.1	45.0 ± 8.4	−11.5	−10.6	+0.9	0.83
**NIH Toolbox: perceived stress**	53.3 ± 5.7	45.7 ± 4.0	45.1 ± 8.3	−7.6	−8.2	−0.6	0.28
**Chronic Pain Acceptance: activity engagement**	45 ± 7.2	45.4 ± 10.9	50.8 ± 8.8	+0.4	+5.8	+5.4	0.39
**Chronic Pain Acceptance: pain willingness**	16 ± 9.1	27.6 ± 5.8	36.2 ± 5.4	+11.6	+20.2	+8.6	1
**Self-Efficacy for Managing Chronic Disease**	6.0 ± 1.8	8.3 ± 0.3	7.5 ± 1.8	+2.3	+1.5	−0.8	0.52
**WHO Disability Assessment Schedule**	51.8 ± 8.6	47 ± 5.1	45.2 ± 10.0	−4.8	−6.6	−1.8	0.16
**EuroQol 5-Dimensions 5-Levels**	0.79 ± 0.04	0.91 ± 0.08	0.88 ± 0.07	+0.12	+0.09	−0.03	0.58
**EuroQol 5-Dimensions 5-Levels: Visual Analog Scale**	65 ± 21.2	80.4 ± 12.3	86.4 ± 5.1	+15.4	+21.4	+6.0	0.76

The mean ± SD values are provided for each measure and each study time point. Kendall’s W test informed on effect sizes. Small effect: 0.1 ≤ W < 0.3; moderate effect: 0.3 ≤ W < 0.5; large effect: W ≥ 0.5.

The Headache Impact Test^49^ scores improved (decreased) from baseline to weeks 3 and 15 with the largest improvements observed in patients with craniofacial FD. Using the painDETECT scale,^50^ at week 0, 2 patients screened positive for neuropathic-like pain (Score: ≥19), 2 patients screened unclear (Score: 13-18), and 1 was negative for neuropathic-like pain (Score: ≤12). The majority of patients at weeks 3 (*N* = 4) and 15 (*N* = 5) were either unclear or negative for neuropathic-like pain. On the Central Sensitization Inventory,^51^ 1 patient had severe central sensitization presentation (Score: ≥50), 2 had moderate presentation (Score: 40-49), and 2 had mild presentation (Score: 30-39). By week 15, all patients were in the moderate to subclinical range for central sensitization.

On questionnaires assessing maladaptive pain-related thoughts and behaviors (ie, the Pain Catastrophizing Scale^52^ and Fear of Pain Questionnaire^53^) robust decreases were quantified at weeks 3 and 15. These findings are in accord with self-reported improvements in anxiety (PROMIS Anxiety scale; T-score metric),^54^ which decreased an average −8.6 at week 3 and −15.2 at week 15. Symptoms of depression (PROMIS Depression scale) and perceived stress (NIH Toolbox Perceived Stress measure) were 2 domains that represented persistent emotional health challenges experienced by patients. Here, 3 participants exhibited a rebound at week 15 follow-up for both depression and stress.

**Figure 5:**
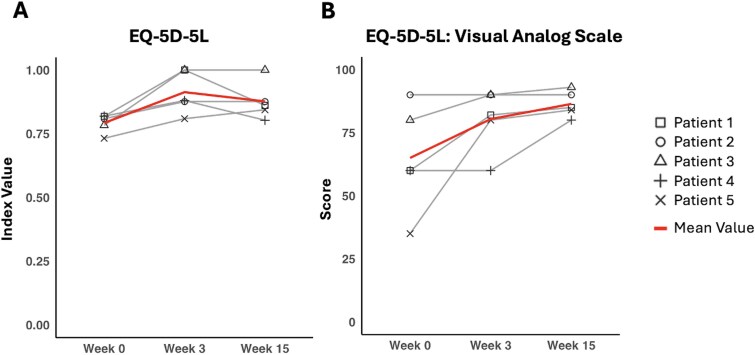
Patient responses to the EuroQol-5Dimensions-5Levels (EQ-5D-5L) at weeks 0, 3, and 15. (A) The EQ-5D-5L index values, which reflects health-related quality of life across mobility, self-care, usual activities, pain/discomfort, and anxiety/depression domains, showed overall improvements at weeks 3 and 15, but some rebound occurring at week 15 in 2 patients. (B) Based on the EQ-5D-5L-visual analog scale, an improvement in perceived overall health was seen for most patients, with patient 2 reporting a consistent level of overall health from weeks 0 to 15.

Patient responses on the Chronic Pain Acceptance and Self-Efficacy for Managing Chronic Disease questionnaires indicated improvements in participating in daily life activities, particularly during pain episodes, improvements to managing their pain in a healthy manner (Table 1). These finding may relate to the improvements in quality of life. The overall health status as measured by the EQ-5D-5L^55^ (index values) showed improvements by an average 0.12 and 0.09 at week 3 and 15, respectively, while EQ-visual analog scale (VAS) ratings increased by an average 15.4 points and 21.4 points at weeks 3 and 15, respectively (Figure 5). Findings on the EQ-5D-5L paralleled the decreasing trend on the WHO Disability Assessment Schedule (Table 1). Finally, the Page test for monotonicity applied across all patient-reported outcomes identified several measures that had significant decreasing or increasing trajectories across weeks 0, 3, and 15 (Table 2).

**Table 2:** Page test results.

**Outcome measure**	**Direction**	**Page test *p*-value**
**Brief Pain Inventory: average pain**	Decreasing	.007^*^
**Brief Pain Inventory: pain right now**	Decreasing	.02^*^
**Brief Pain Inventory: least pain**	Decreasing	.04^*^
**Brief Pain Inventory: worst pain**	Decreasing	.001^*^
**Brief Pain Inventory: pain severity composite score**	Decreasing	.001^*^
**Brief Pain Inventory: pain interference composite score**	Decreasing	.007^*^
**PainDETECT**	Decreasing	.001^*^
**Central Sensitization Inventory**	Decreasing	.007^*^
**Headache Impact Test**	Decreasing	.02^*^
**PROMIS: Pain behavior**	Decreasing	.001^*^
**Pain Catastrophizing Scale**	Decreasing	.0001^*^
**Fear of Pain**	Decreasing	.04^*^
**PROMIS: anxiety**	Decreasing	.001^*^
**PROMIS: depression**	Decreasing	.02^*^
**NIH Toolbox: perceived stress**	Decreasing	.14
**Chronic Pain Acceptance: activity engagement**	Increasing	.04^*^
**Chronic Pain Acceptance: pain willingness**	Increasing	.14
**Self-Efficacy for Managing Chronic Disease**	Increasing	.04^*^
**WHO Disability Assessment Schedule**	Decreasing	.14
**EuroQol 5-dimensions 5-levels**	Increasing	.04^*^
**EuroQol 5-dimensions 5-levels: Visual Analog Scale**	Increasing	.007^*^

^*^ *p* < 0.05.

### Patient-specific alteration during treatment

Patients 1, 2, and 5 reported alterations in their use of their respective pain medications or assistive devices. Patient 1 tapered the use of propranolol from 160 mg daily to 40 mg twice daily, which improved her fatigue without increased pain flare-ups. She also reduced the frequency of rescue medications (ubropgepant, rimegepant, and naproxen) during the 3-wk treatment period. At week 3, patient 2 discontinued use of THC edibles. At week 3, patient 5 was able to walk for more than 30 min without using a cane. Reductions of pain medication and use of assistive devices was initiated by patients but managed and supervised by a pain physician.

### Physician Global Assessment

**Figure 6:**
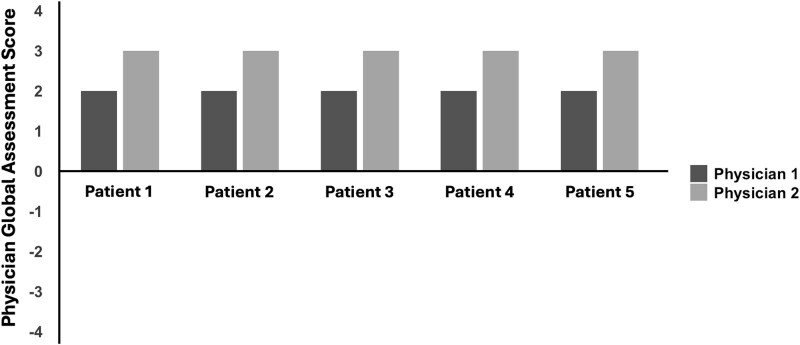
Physician Global Assessment score at week 3. The Physician Global Assessment was independently completed by 2 physicians. Both physicians noted improvements in the patients’ status at week 3 relative to week 0.

Two physicians (physicians 1 and 2) independently evaluated each patients’ pain, emotional, and functional status using the PGA (−4 to +4 scale) at week 0 and 3 (Figure 6). Physician 1 rated all 5 patients as +2 or improved. Physician 2 rated all 5 patients as +3 or much improved.

### Laboratory data including bone turnover markers

Changes in laboratory measures, including calcium, phosphorus, albumin, CTX, and ALP, are summarized in Table S2. No consistent changes were observed over the treatment period.

## Discussion

This prospective, single-site, pilot trial demonstrated the initial feasibility and clinical utility of a 3-wk IIPT program in a small cohort of young adults diagnosed with FD/MAS. Consistent with prior work, personalized pain treatment regimens were administered in individual or group settings by an interdisciplinary team of a pain physician, psychologists, physical therapists, and occupational therapists.^40^^,^^56^^,^^57^ A biopsychosocial treatment approach was particularly relevant to the current patients to support participation in valued activities while learning to self-manage symptoms in the context of elevated fear of movement, pain, or injury. All patients indicated a high-level of acceptability, appropriateness, and feasibility of an IIPT intervention indicating that this therapeutic model was well-received. Relative to week 0 (treatment admission), a trend of reduced pain severity, pain interference, and pain catastrophizing as well as improvements in emotional health (ie, less severe depression and anxiety), physical health, and overall quality of life were observed at week 3 (treatment discharge) and week 15 follow-up. Reductions in pain catastrophizing and pain interference were notable, as these findings may indicate a shift in how coping skills learned in the IIPT program altered the way patients thought about their pain in more supportive ways to help to decrease the impact on daily functioning. Questionnaire-based, patient reported outcomes informing on pain or pain-related elements, such as emotional well-being, were corroborated by PGAs and open-ended feedback obtained from each patient at treatment discharge. Finally, several patients reduced their use of pain medications during the 3-wk treatment period under appropriate supervision by the pain physician.

Many prior FD/MAS investigations have studied pharmacological approaches, such as denosumab, bisphosphonates, and other therapies,^7^^,^^23^^,^^24^^,^^58–64^ with primary outcomes appropriately focused on quantifying the efficacy of these treatments on FD lesion activity as measured by imaging or bone turnover markers. When evaluated, pain and disability-related outcomes have generally been included as secondary endpoints rather than primary indicators of therapeutic response. Although retrospective analyses have suggested that denosumab may alleviate pain in FD/MAS,^65^ prospective trials using denosumab did not reveal significant changes in pain measures (ie, BPI) even though robust reductions in FD lesion activity were evident.^66^ While the underlying mechanisms causing this disconnect are not fully understood, case reports and preclinical studies suggest that RANKL inhibition can reduce peripheral nociceptive drive via suppression of osteoclast-mediated extracellular acidosis^24^^,^^67^^,^^68^; however, maladaptive centralized or nociplastic processes are likely also active in patients with FD/MAS, facilitating persistent pain and disability that is not impacted by treatments that primarily target skeletal pathology.^6^^,^^17^^,^^38^ This working hypothesis is also consistent with the biopsychosocial model of chronic pain, which emphasizes the dynamic interactions of biological (central or peripheral), psychological, and social factors that contribute to pain phenotypes in any clinical conditions. Consistent with this framework, we observed no consistent changes in bone turnover markers over the treatment period despite improvements in pain and functional outcomes. These findings align with prior observations that pain in FD/MAS is not associated with lesion activity.^17^ How therapies such as denosumab, which effectively target skeletal pathology, can be integrated with personalized biopsychosocial pain therapeutic approaches may represent a novel and comprehensive treatment strategy in FD/MAS. The current findings suggest that IIPT and biopsychosocial models of care may fill an important gap, in a complimentary manner, toward improving pain, disability, and quality of life in FD/MAS, especially if pharmacologic or surgical approaches provide incomplete or inconsistent relief. The biopsychosocial framework underlying IIPT may also have broader applicability to other rare bone diseases (eg, OI and fibrodysplasia ossificans progressiva) in which chronic pain is a prominent and undertreated feature.^7^^,^^69^

We note several limitations for the current trial which may be addressed in future investigations. This study enrolled a small cohort of patients, utilized a 3-wk treatment period, and lacked a control arm in the study design. Fibrous dysplasia is a rare disorder; the small sample size and clinical heterogeneity of the cohort limit interpretation of treatment outcomes. While this patient cohort reflects the real-world spectrum of FD/MAS, it prevents subgroup analyses. As a pilot study, the analyses are exploratory and intended to assess feasibility and describe preliminary trends. While the current preliminary trial supports the utilization of a non-pharmacological approach to reduce pain and improve functioning in FD/MAS, additional controlled studies involving larger cohort sizes, longer treatment duration (eg, 4-6 wk; some patients noted the treatment period was short) and longer-term follow-up (eg, 52 wk) are critical for further deciphering the clinical utility and durability of IIPT in FD/MAS. Furthermore, future studies involving a larger cohort of FD/MAS patients may identify certain phenotypic characteristics associated with greater clinical benefit from IIPT. Future trials may benefit from incorporating comparative groups; although comparison with a similar historical FD/MAS cohort (Figure S1) provides contextual support that the observed improvements are likely not solely attributable to natural symptom fluctuation, this comparison is not a substitute for a prospective control group. Assessing pain severity, emotional and physical health, and quality of life among patients receiving pharmacological therapy (eg, denosumab) + biopsychosocial treatment vs those receiving solely pharmacological therapy could better determine the value of the biopsychosocial approach as an adjunctive treatment. Follow-up studies should evaluate the effectiveness of this treatment in patients with FD/MAS with more severe disease, particularly those that who are non-ambulatory, as well as pediatric and older adult FD/MAS cohorts, as these sub-populations may require a unique set of therapeutic strategies to reduce pain and enhance the patients’ functioning. Future larger-scale trials should incorporate measurement of candidate pain biomarkers, including neurotrophins and inflammatory cytokines, to determine whether IIPT produces measurable (neuro-)biological changes alongside clinical improvements.

In conclusion, this pilot study provides preliminary support for the feasibility, acceptability, and safety of a biopsychosocial pain treatment approach for patients with FD/MAS. This investigation highlights the complex and multidimensional nature of pain in FD/MAS and offers further support for the use of an interdisciplinary approach to alleviate pain and improve functioning and quality of life in individuals with this disease.

## Supplementary Material

FD_IIPT_SupplementalMaterial_061725_clean_ziag107

## Data Availability

Restrictions apply to the availability of some, or all data generated or analyzed during this study to preserve patient confidentiality. The corresponding author will on request detail the restrictions and any conditions under which access to some data may be provided.
