# Supplementary material for: Intensive, interdisciplinary pain treatment in fibrous dysplasia/McCune–Albright syndrome
Source: JBMR Plus. 2026 Jun 29;10(8):ziag107. doi: 10.1093/jbmrpl/ziag107 (PMC13395099; doi:10.1093/jbmrpl/ziag107)
Supplement: FD_IIPT_SupplementalMaterial_061725_clean_ziag107 [file fd_iipt_supplementalmaterial_061725_clean_ziag107.docx]

**
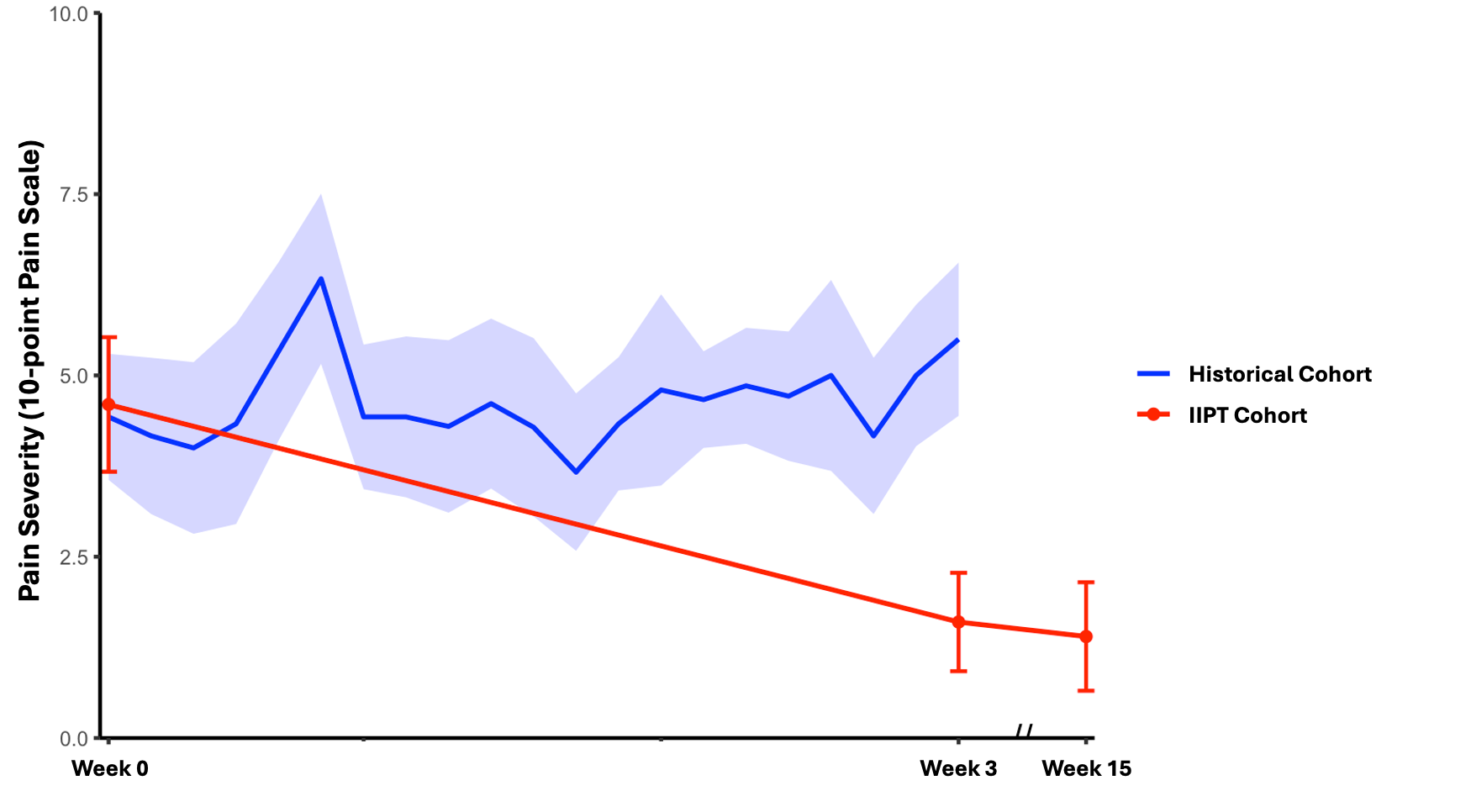
**

**Supplemental Figure 1. Patient-Reported Pain Severity Compared with a Historical Cohort.** Mean patient-reported pain severity (0–10 scale) in the current Intensive Interdisciplinary Pain Treatment (IIPT) cohort (red) is shown at treatment admission (week 0), discharge (week 3), and mid-term follow-up (week 15). Pain severity decreased by 3.0 points at week 3 and 3.2 points at week 15 relative to week 0. Error bars represent standard error for the IIPT cohort. The historical FD/MAS cohort (blue; n = 7), comprised of patients who would have met parallel eligibility criteria for the current study (pain >4/10), reported daily pain ratings over a three-week period; mean values are shown with standard error represented by the shaded region. These patients did not receive IIPT; some intermittently used pain medications (NSAIDs and opioids), and one received denosumab without pain improvement. In contrast to the IIPT cohort, the historical cohort showed no meaningful change in pain severity over time.

*Alt text: A line graph comparing pain severity over time between two cohorts. The blue line (Historical Cohort) with shaded 95% confidence interval fluctuates around 4–6 on a 10-point pain scale from Week 0 through the 3-week follow-up period, showing no clear downward trend. The red line (IIPT Cohort) starts near 5 at Week 0, then declines steeply to approximately 1.5 at Week 3 and Week 15, with error bars shown at those time points.*

**Supplemental Table 1: Physician Global Assessment**

| **PHYSICIAN GLOBAL ASSESSMENT (PGA)** | | |
| --- | --- | --- |
| **Patient**:  **Investigator**: | | **Date of Treatment**:  **Date of Assessment**: |
| **Please rate the overall response to treatment considering:**   1. **Change in impairment** 2. **Change in performance / function based on objectives / goals of treatment** 3. **Change in activity and participation restrictions**   **All physician global assessment ratings will be performed with input from the physical examination, interim medical history, any available videotape of upper limb and/or gait function, assessment of objectives of treatment, changes in activity and participation restrictions, use of splints/braces & assistive devices, and patient interviews.** | | |
| ***How would you rate the subject’s overall response to treatment?*** | | |
| **+ 4** | Markedly Improved | |
| **+ 3** | Much Improved | |
| **+ 2** | Improved | |
| **+ 1** | Slightly Improved | |
| **0** | No Change, in her functioning | |
| **- 1** | Slightly Worse | |
| **- 2** | Worse | |
| **- 3** | Much Worse | |
| **- 4** | Markedly Worse | |

**Supplemental Table 2: Cohort Characteristics and Laboratory Markers**

|  | **Patient 1** | | **Patient 2** | | **Patient 3** | | **Patient 4** | | **Patient 5** | |
| --- | --- | --- | --- | --- | --- | --- | --- | --- | --- | --- |
| **Sex** | F | | F | | F | | F | | M | |
| **Age** | 24 | | 27 | | 21 | | 25 | | 30 | |
| **BMI** | 20.6 | | 21.1 | | 19.9 | | 22.2 | | 39.2 | |
| **Diagnosis** | PFD | | MFD | | MAS | | PFD | | MFD | |
| **Involved Skeletal Region** | Craniofacial | | Craniofacial | | Craniofacial, axial, appendicular | | Craniofacial, appendicular | | Appendicular | |
| **SBS** | 7 | | 7 | | 40 | | NA* | | 8 | |
| **# of fractures** | 0 | | 0 | | 4 | | 3 | | 0 | |
| **Previous Surgeries** | none | | craniofacial recontouring x 3 | | R intramedullary nail  L intermedullary rod | | L femoral osteotomy  L intermedullary rod and plate | | intramedullary nail with allograft  repeat allograft  intermedullary rod placement | |
|  | **Week 0** | **Week 3** | **Week 0** | **Week 3** | **Week 0** | **Week 3** | **Week 0** | **Week 3** | **Week 0** | **Week 3** |
| **Calcium** | 9.3 | 9.6 | 10 | 10 | 9.6 | 9.1 | 9.2 | 9.2 | 9.4 | 9.6 |
| **Phosphorus** | 4.3 | 4.7 | 3.1 | 3.6 | 3.6 | 2.7 | 2.1 | 3.7 | 3.4 | 3.9 |
| **Albumin** | 4.5 | 4.6 | 4.6 | 4.6 | 4.5 | 4.2 | 4.6 | 4.5 | 4.5 | 4.6 |
| **Alkaline Phosphatase** | 48 | 54 | 62 | 67 | 206 | 242 | 91 | 89 | 68 | 60 |
| **CTX** | 155 | 173 | 280 | 167 | 980 | 1094 | 606 | 941 | 298 | 229 |
| **P1NP** | 40 | 49 | 51 | 50 | 286 | 289 | 121 | 116 | 45 | 69 |

***Patient had only had MRIs; no nuclear bone scan was available. Patient has known lesions in the skull, mandible, left tibia, left femur, and right femur.

**Supplemental Table 3: Quotes from Open-Ended Interview Responses**

| **Patient**  **ID** | **What were the most helpful parts of this program?** | **What parts of the program do you wish had been different?** | **What were the most important things that you learned from the program?** |
| --- | --- | --- | --- |
| **1** | “the specialists were operating as a team”  “the overall lifestyle evaluation”  “the education” | “having a neurologist involved [could help]” | “even though I can't work towards curing my CFD, I can work towards living the life I want to live” |
| **2** | “the education on chronic pain that helped make my life make just a little bit more sense”  “learning how to pace myself” | “bringing in a neurologist…would have enhanced the program” | “my pain is not going to go away but how I choose to live my life and address it can make improvements” |
| **3** | “OT…came up with really helpful plans and strategies and techniques for activities in my everyday life” | “the timeslot at the end of the day” | “the biopsychosocial model they use…having it all together was really helpful” |
| **4** | “the psych counselor was incredibly helpful [to develop] strategies for myself”  “home exercises and modifications” | “I wish…group sessions were the first sessions instead of last sessions [of the day]” | “now I have a whole list of coping strategies”  “their recommendations…were the most helpful” |
| **5** | “psychology”  “I got a lot of the help and push I needed to confront my mental health” | “I would like at least another week” | “that whole model of how pain [is created] and that that could be dealt with” |

**Case Descriptions:**

***Patient 1 Overview:*** Patient 1 is a 24-year-old female with polyostotic fibrous dysplasia (FD) involving the right orbit, maxilla, zygoma, and mandible. She was diagnosed at 16 years of age by an orthodontist due to a disparity in mandible size. The patient has no history of oral and maxillofacial surgery. Starting at age 17, she received zoledronate every six months to reduce lesion activity and manage pain. The patient noted an improvement in her pain, which typically flared up again several weeks before each infusion. She was using gabapentin, carbamazepine, rimegepant, ubrogepant, naproxen, and propranolol for pain management at the time of treatment admission.

*Patient 1 Baseline History and Clinical Assessment:* At admission, Patient 1 reported constant right-sided trigeminal pain over maxillary and mandibular nerve distributions with intermittent electric shock-like pain 15 to 20 days per month. Pain was triggered by temperature changes, position changes, and emotional stress. She also experienced mirror-image trigeminal pain on the left side which was less severe and bilateral intermedius neuralgia and ear pain. The patient reported frequent migraines that contributed to sleep disruptions, daytime fatigue and poor attention. Her menarche started at 13 years, menstrual cycles are regular, but they trigger the trigeminal neuralgia pain during the premenstrual period. Additionally, she reported arthritis of the lower back and pelvis. The physical examination showed right-sided facial asymmetry consistent with FD but was otherwise normal. The general musculoskeletal and neurological exam was within functional limits.

She maintained a high baseline activity level, running 3 to 5 miles on most days and attending Pilates twice weekly. The patient reported difficulty with position changes, cervical rotation to the affected side, stair navigation, and concentration during sedentary work tasks due to pain. Physical tasks aggravated symptoms at work, and she reported avoiding painful activities due to concerns that they would cause harm, a prolonged time of rest for recovery, or be too difficult to manage. She reported a tendency to use “all or nothing” thinking styles which resulted in “pushing through” pain, leading to fatigue consistent with a boom-bust activity pattern^^[[1]](#footnote-1)^^.

Patient 1 noted that her diagnostic and treatment course had previously been prolonged and disruptive, which contributed to emotional distress and impairment. She reported that initially her headaches were attributed to a premorbid diagnosis of anxiety. At 16, she developed depression, which she attributed to the year-long series of hospital appointments surrounding her FD diagnosis. She was previously treated with psychotherapy and SSRIs. At baseline evaluation, her depression was in complete remission. She endorsed anxious thought patterns consistent with some perfectionistic tendencies. She described not knowing other people with FD and undergoing many of her surgical treatments and recoveries as lonely and isolating in the past. She described a current strong social support network and is well-connected with trusted providers.

*Patient 1 Individualized Treatment Plan:* The treatment plan developed for Patient 1 focused on functional restoration, pacing, and coping with pain and fatigue. Goals included increasing knowledge about chronic pain, practicing pacing techniques to avoid boom-bust cycling, improving sleep hygiene, and gradually exposing oneself to pain or symptom triggers. Treatment emphasized strengthening and endurance within her existing exercise routine and pain neuroscience education, targeted activity modification at work and self-awareness of activity tolerance, and tailoring bio-behavioral strategies for pain and emotional regulation, (e.g., normalizing emotional experience with rare chronic illness, and cognitive diffusion techniques based in Acceptance and Commitment Therapy (ACT) to address perfectionism). She received an individualized pain flare coping plan utilizing active coping strategies (i.e., distraction, relaxation, movement, and stretching) in combination with helpful passive coping strategies (i.e., ice and medications). The patient’s medication regimen was also reviewed in the context of daily vitals and activity to maximize benefit and reduce unintended side effects (e.g., fatigue or rebound headache).

***Patient 2 Overview:*** Patient 2 is a 27-year-old female with monostotic FD affecting the left maxilla. She was diagnosed at age 23 and had undergone three contouring procedures to the maxilla to improve facial asymmetry. Her medical history included non-specific hyperthyroidism that was treated with methimazole until it resolved at age 22. She was not on any medications for FD treatment but managed her anxiety with alprazolam and used ibuprofen and unprescribed tetrahydrocannabinol (THC) edibles when experiencing pain.

*Patient 2 Baseline History and Clinical Assessment:*The patient described constant left maxillary pain that worsened in the previous year. Pain was described as “pulsating,” “shooting,” and “lingering,” sometimes accompanied by nose bleeds, congestion, and swelling in the left cheek. She also reported daily electric shock-like pain in the left palate and weekly ocular migraines with distorted vision and photophobia. Her menarche started at 13 years, and she is currently on birth control. Examination showed left-sided facial asymmetry consistent with FD but was otherwise normal. Her general musculoskeletal and neurological exam was unremarkable. The patient was independent in mobility and activities of daily living. Physical therapy evaluation showed normal strength, balance, and endurance.

The patient endorsed symptoms of anxiety, which were worsened by pain and disease-related fears as well as around disease progression and changes in appearance. She endorsed mild obsessive-compulsive tendencies as well as a history of medical trauma related to the process of being diagnosed with FD and to prior surgeries. She also described anxiety-related appetite suppression. Additionally, the patient also reported depressed mood, fatigue, irritability, mild anhedonia, and wishes to be pain-free; however, depressive symptoms were described as situational rather than indicative of a depressive disorder.

Pain frequently interfered with daily functioning including work hours and contributed to delayed sleep onset, fatigue, and diminished memory. She worked full-time in person and exercised regularly, typically through walks or calisthenics, 4 to 5 times per week. She reported a pattern of pushing through pain at work, followed by fatigue and withdrawal on weekends. Although the patient described close relationships with friends and family, she occasionally canceled social plans during pain flares and when fatigued from a week of work while dealing with pain. She endorsed THC use 4 nights per week for pain and anxiety management. During her rehabilitation treatment she reduced the frequency of her rescue medication of alprazolam and THC edibles.

*Patient 2 Individualized Treatment Plan:* The treatment plan focused on pacing and coping strategies. Goals included energy conservation, improved sleep hygiene, and graded exposure to pain triggers. Physical therapy focused on improving strength and endurance, while occupational therapy addressed activity modification and time management. Psychological therapy taught cognitive restructuring and acceptance-based techniques to address anxious thoughts and strategies for emotional regulation and pain management.

***Patient 3 Overview:*** Patient 3 is a 21-year-old female with McCune-Albright syndrome, with FD lesions affecting the skull, ribs, spine, pelvis, both femurs, right tibia, and right foot. She was diagnosed following menarche at age 6. She was placed on puberty blockers until age 9 and experienced a regular menstrual cycle. She was diagnosed with FD at age 6 because of the pain in her lower extremities. She fractured both femurs in childhood (right femur at age 9, left femur at age 16) requiring intramedullary rod placement and screw fixation. She was taking daily calcium and vitamin D supplements and used heat, ice, and stretching for pain management at the time of admission. The patient recently graduated from college and was starting a job in healthcare. She was not taking any medications except for occasional over-the-counter acetaminophen or NSAIDs.

*Patient 3 Baseline History and Clinical Assessment:*The patient reported persistent right hip pain and intermittent right knee pain, particularly with weight-bearing or high-impact activities. Hip pain was aggravated by walking and high-impact activities, sometimes lasting one to two weeks during flares. Knee pain was described as burning and tingling around the patella and anterior thigh and was more prominent in the preceding month. She also endorsed intermittent right-sided ribs and mid-back pain, making it difficult to lie on her right side. She had a history of right-sided rib fractures. She avoided running, jumping, and other high-impact activities due to fear of bone injury. She swam once a week and attended Pilates twice a week but described decreased endurance and occasional pain flares with changes in her activity level. She was independent with all activities of daily living but occasionally experienced periods of “crashing” following increased physical activity. She reported stiffness in her lower back after prolonged sitting and avoided sleeping and weight-bearing on her right side due to rib pain. Physical examination of the musculoskeletal and neurological systems was unremarkable except for mild scoliosis and ligamentous laxity. She reported difficulty standing and lifting patients during her hospital nursing observership. The patent endorsed a generally positive mood, with transient low mood and anxiety related to pain or injuries. She described feeling well-supported by her parents who became strong advocates for her FD treatment with providers when she was a child and adolescent. She noted that moving away from specialty and trusted providers for college resulted in difficulties obtaining needed medical care for unexpected FD-related injuries, which contributed to changes in mood. Patient 3 described strong family and peer support.

*Patient 3 Individualized Treatment Plan:* The treatment plan focused on functional restoration and preventing activity avoidance. Goals included graded exposure to pain triggers, pain neuroscience education, pacing strategies to avoid boom-bust activity cycles, and improved endurance. Physical therapy emphasized progressive general strengthening and conditioning to improve her endurance for activities and her occupation, as well as body mechanics and postural education for safe patient lifting. Additionally, it included practice and education on safe participation in recreational activities. Occupational therapy targeted energy conservation and adaptation for lifting. Psychological therapy utilized CBT for pain and chronic illness, focused on pain and chronic illness education, cognitive restructuring, active coping, and advocacy skills as a young adult in the medical system.

***Patient 4 Overview:*** Patient 4 is a 25-year-old female with polyostotic FD affecting the left femur, left tibia, mandible, and occipital bone. She was diagnosed at age 5 and sustained multiple fractures in childhood, requiring several orthopedic procedures, including rod placement in the left tibia and left distal femoral osteotomy with rod and plate fixation. She had a history of follicular thyroid cancer at age 16 that was treated with total thyroidectomy and was taking levothyroxine. The only additional medication at the time of treatment admission was ibuprofen used as needed during pain flares.

*Patient 4 Baseline History and Clinical Assessment:*Patient 4 presented with intermittent pain in the left knee and shin down to the big toe and left anterior thigh pain and numbness in the distribution of the superficial peroneal nerve. These symptoms increased with weightbearing and physical activity, including Pilates, lunges, prolonged walking, and sitting, which was consistent with left piriformis syndrome. She experienced fatigue, mobility difficulty, and reduced socialization due to pain, and these difficulties were associated with frustration and anger about her leg pain and functional limitations. However, she denied depression symptoms. She used a right heel lift but avoided using a cane due to discomfort about negative perception by others and symptoms of social anxiety, despite prior benefit from assistive devices. She had difficulty maintaining prolonged seated postures during work and preferred to use a reclining chair or a standing desk. She endorsed non-restorative sleep and low-to-normal energy levels. On physical examination, her strength and mobility were largely intact, except for the left gluteus medius and gluteus maximus. Weakness and gait were significant in a compensated left Trendelenburg position. She also exhibited an apparent leg length discrepancy of 2.5 cm (left leg shorter), which contributed to her gait mechanics and caused mild difficulty with single-leg stance on the left. She engaged in regular physical activity, including hiking, skiing, swimming, and Pilates, but sometimes canceled exercises or other activities when the pain was severe. She also reported a history of childhood trauma and obsessive-compulsive behaviors around cleanliness. She engaged in long-term supportive psychotherapy, meeting monthly at the time of treatment admission, to address the psychological difficulties she experienced associated with pain/functional difficulties as well as those that were independent of her pain experiences. She described strong and supportive relationships.

*Patient 4 Individualized Treatment Plan:* The treatment plan developed for Patient 4 focused on functional restoration, active pain management, addressing maladaptive coping, and addressing cognitive distortions about physical appearance related to her surgery scars and gait. Goals included improving gait mechanics and improving activity tolerance and pacing. Physical therapy emphasized addressing her gait, leg length discrepancy, and piriformis syndrome. Pain neuroscience and biomechanics education related to her presentation was emphasized to enhance self-management. The patient received an over-the-counter shoe lift to address her leg length discrepancy and exercises to address her piriformis syndrome; this improved her gait and symptoms. Local orthopedic and physical therapy referrals were provided to address biomechanical concerns further. Occupational therapy targeted pacing skills and graded activity participation. Psychosocial therapy focused on pain and chronic illness psychoeducation, CBT for pain and chronic illness, addressing frustration, self-criticism, and social anxiety through cognitive restructuring and active coping strategies, and the use of ACT techniques to support acceptance and normalization of chronic illness.

***Patient 5 Overview:*** Patient 5 is a 30-year-old male with monostotic FD in the left tibia. He was diagnosed at age 12. He had undergone three orthopedic surgical procedures to treat his FD-related pain, including intramedullary nail placement with allograft, repeat bone grafting, and intermedullary rod placement. He had type 1 diabetes managed with self-administered insulin but did not have a diagnosis of peripheral neuropathy. He had recently discontinued tramadol before admission.

*Patient 5 Baseline History and Clinical Assessment:*The patient reported mid-tibial pain that worsened with weightbearing, walking, and high-impact activities. The site of the prior allograft was tender upon palpation. At the time of admission, he used a cane during ambulation to relieve pain as well as provide balance and support. He described his pain as progressive and feared disease progression. Before his most recent surgery, pain was severe enough that he requested amputation as a pain management option, and he described a history of suicidal ideation during periods of uncontrolled and unmanageable pain. The physical examination revealed well-healed surgical scars and tenderness in the mid-leg. His musculoskeletal and neurological exam was otherwise unremarkable. He reported difficulty standing longer than 10 to 30 minutes and walking more than 1 km. He typically used a single-point cane for mobility in the community. He avoided impact activities, such as running, jumping, or biking, due to a fear of pain flare-ups and injury. He discontinued regular exercise due to pain and difficulty walking to and from the gym. He presented with global deconditioning and muscle weakness due to sedentary lifestyle and pain avoidance. Pain impacted his ability to function. Although he was independent with all activities of daily living, he endorsed increased fatigue and non-restorative sleep and described periods of “all or nothing” activity, most especially within academic performance, that often led to overexertion and pain flares. The patient described many periods of his life when pain and mood difficulties have interfered with his ability to attend school and go to work. Although he described a supportive friend group and close relationships with family, pain would also interfere with his ability/willingness to socialize with friends and family. He endorsed chronic post-traumatic stress disorder (PTSD) symptoms related to childhood trauma and pain-related experiences, recurrent major depressive disorder and generalized anxiety along with traits consistent with autism spectrum. Although these psychological struggles arose independent of pain, they were ultimately exacerbated by his difficulties with pain and functioning.

*Patient 5 Individualized Treatment Plan:* The treatment plan focused on improving function, addressing maladaptive coping, and reducing pain-related disability. Physical therapy goals included graded activity with general strengthening and conditioning through a daily home exercise program to improve endurance and independence with community mobility. Treatment also targeted the discontinuation of mobility aids, as well as balance and pain neuroscience education. Occupational therapy supported pacing strategies to reduce boom-bust cycles and enhance social and academic participation. Psychological interventions focused on providing the patient with psychoeducation on possible psychological diagnoses under consideration and describing typical psychological treatments used to address the symptoms he reported, using CBT techniques to address depression and anxiety, offering strategies for PTSD symptom management, as well as developing emotional regulation and active coping strategies. Psychotherapy referrals were also provided during the study to ensure that trauma-focused treatment was pursued post-study. Additionally, the patient’s management of type 1 diabetes was discussed and monitored. During the last 10 days of treatment his blood sugar stabilized.

**List of Clinical Questionnaires:**

Brief Pain Inventory – Captures pain severity and pain interference on activities of daily living in the past 7 days

PainDETECT - Screens for neuropathic pain features to help distinguish neuropathic from nociceptive pain

Central Sensitization Inventory - Symptoms associated with central sensitization

Headache Impact Test - Measures the impact of headaches on daily life

PROMIS: Pain Behavior - Assesses the frequency and impact of pain-related behaviors in the past 7 days

Pain Catastrophizing Scale - Measures maladaptive thoughts and feelings about pain

Fear of Pain - Assesses fear and avoidance beliefs related to pain and potential injury

PROMIS: Anxiety - Severity of anxiety symptoms in the past 7 days

PROMIS: Depression - Severity of depressive symptoms in the past 7 days

NIH Toolbox: Perceived Stress - Informs on the presence of internal, emotional challenges, feeling overwhelmed in the past month

Chronic Pain Acceptance - Measures willingness to experience pain without excessive attempts to control it (pain willingness) and engagement in life activities despite pain (activity engagement)

Self-Efficacy for Managing Chronic Disease - Assesses self-confidence in ability to manage challenges associated with chronic illness

WHO Disability Assessment Schedule - Evaluates disability and functional status across life domains (cognition, mobility, self-care, relationships, household activities, school/work activities, and participation in society)

EuroQol - 5 Dimensions - 5 Levels - Assesses health-related quality of life across five dimensions (mobility, self-care, usual activities, pain/discomfort, and anxiety/depression)

EuroQol - 5 Dimensions - 5 Levels: Visual Analog Scale - self-rated overall health on a quantitative visual analog scale

1. The boom-bust pattern is a maladaptive behavioral process related to ‘good days’ and ‘bad days’ with respect to pain, pain-related symptoms (i.e., fatigue), and activity. On days when a patient may experience little to no pain, for example, or when there is an activity that is especially important to them, individuals may function without stopping or pacing – a boom. This then results in increased pain, fatigue, and recovery with rest and reduced activity – the bust. Over time, this reduces the level of functioning and creates a cyclical pattern of pain flares and relatedly, activity limitations. [↑](#footnote-ref-1)
